# “I Could Stop and Breathe”: Early Implementation Results of a Short-Term Care Coordination Model for Children with Medical Complexity

**DOI:** 10.5334/ijic.8975

**Published:** 2025-03-26

**Authors:** Stephanie Hodgson, Ashleigh Griffiths, Christophe Lecathelinais, Camilla Askie

**Affiliations:** 1 Children, Young People & Families Services, Hunter New England Local Health District, Newcastle, Australia; 2 Hunter New England Population Health, Hunter New England Local Health District, Newcastle, Australia

**Keywords:** integrated care, care coordination, children with medical complexity, healthcare innovation, health outcomes, co-design

## Abstract

**Introduction:**

Children with medical complexity (CMC) are a vulnerable population with high healthcare utilisation and significant care coordination challenges. This study evaluates the early implementation results of a short-term Care Coordination Model designed to address these challenges within the Hunter New England Local Health District (HNELHD) in New South Wales, Australia. The Model aims to provide an intensive, time-limited “dose” of care coordination, followed by a Maintenance Phase, to improve healthcare use and reduce the coordination burden on families and healthcare staff.

**Description:**

The Model consists of two phases; an Intensive Phase led by a Paediatric Care Coordinator, providing focused support and care planning for 6–12 months, and a Maintenance Phase where care is handed over to a member of a Paediatric Care Coordination Network for ongoing monitoring and support. A pre-post evaluation of hospital utilisation data for the first 20 children enrolled in the Model was conducted, covering six months before and six months after enrolment. Outcomes measured included outpatient appointments, coordinated appointments, inpatient stays, emergency department presentations, and travel distance for care.

**Discussion:**

The early results from the pilot phase indicate promising outcomes. The Intensive Phase of the Model has led to more coordinated appointments, and reduced travel for families. The concept of “coordination respite” emerged as a significant benefit, where families experienced relief from the constant pressures of managing their child’s care. This respite allowed families to regroup, organise, and find the mental space to learn how to better coordinate their child’s care independently. The Intensive Phase provides critical support during the most demanding times, while the Maintenance Phase is positioned to support sustained, long-term assistance.

**Conclusion:**

The early implementation of the short-term Care Coordination Model for CMC in HNELHD shows significant potential. The Model’s intensive, time-limited approach, combined with a Maintenance Phase and a strong focus on family empowerment and Network collaboration, offers a sustainable approach to care coordination. Future research should continue to explore the optimal dose of care coordination that is aligned with the principles of value-based care and further evaluate the Model’s long-term impact, beyond the Intensive Phase.

## Introduction

Children with medical complexity (CMC) represent a vulnerable population at high risk of care fragmentation and poor quality of care [1]. CMC may be defined as children with multiple chronic health conditions that affect multiple organ systems, resulting in functional limitations, high health care utilisation and often the need for medical technologies [2,3,4]. CMC and their families often encounter significant disruptions and challenges in navigating healthcare, including managing frequent hospital visits, aligning appointments with multiple specialists, and navigating varied medical and support service systems [5]. These challenges are compounded by the complexities of coordinating care across multiple services and providers, making it difficult to manage their health effectively. Despite being 1% of the paediatric population, CMC account for over 30% of paediatric healthcare costs [6].

**Figure 1:**
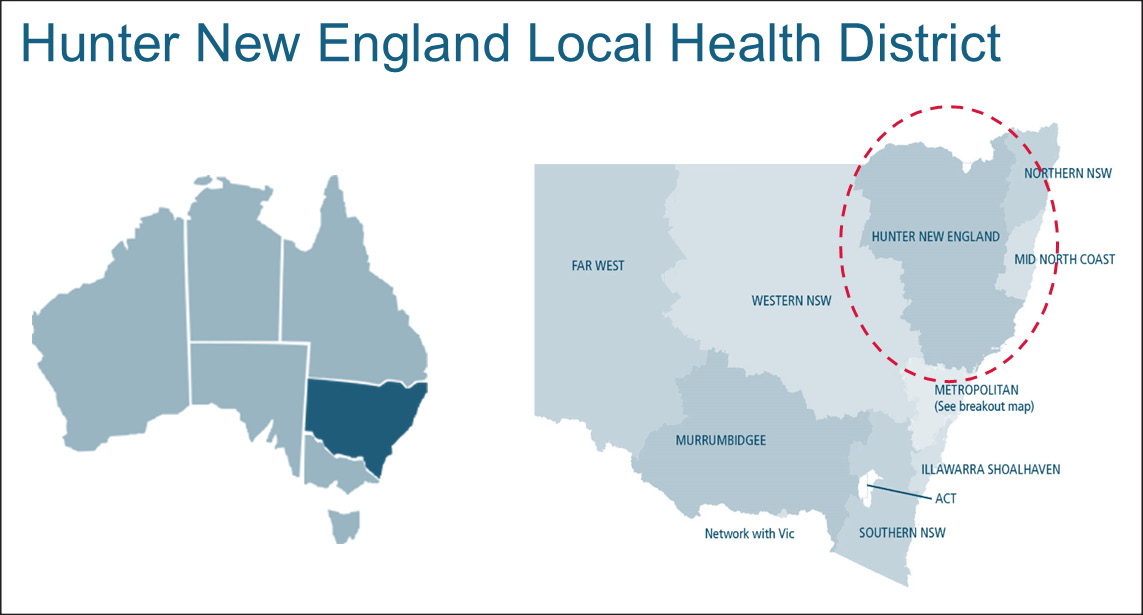
The Hunter New England Local Health District of New South Wales, Australia.

Before 2023, there was no consistent Model for coordinating care for CMC within the Hunter New England Local Health District (HNELHD) in New South Wales (NSW), Australia (Figure 1). Recognising the need for a structured approach, staff within HNELHD undertook a quality improvement project to facilitate the co-design and development of a novel Model of Care (MoC) to improve care coordination for CMC in the district.

Many integrated and coordinated care models described in the literature are specific to adult populations, metropolitan settings, or particular sub-specialties or diseases [7,8,9,10,11]. A noticeable gap exists in research exploring the optimal dose of care coordination, particularly for CMC, given the cumulative and often unsustainable nature of existing care coordination services. This study aims to fill this gap by evaluating the impact of a model that provides intensive, short-term care coordination for CMC.

This paper outlines early results from a pilot of the new Model. We assess changes in hospital utilisation six months before and after enrolling in the Model and share family and staff perspectives on the novel Model. Through this, we aim to contribute to the existing body of knowledge and influence future policies for the care coordination of CMC in Australia.

## Setting

The study took place in HNELHD in NSW, Australia (Figure 1). HNELHD serves a diverse population of over 920,000 individuals, including a significant proportion of Aboriginal and Torres Strait Islander people and residents born overseas. HNELHD includes a major metropolitan centre as well as several large regional centres, smaller rural centres, and remote communities [12]. The paediatric specialty hospital in the district is the John Hunter Children’s Hospital (JHCH), located in Newcastle. JHCH provides specialised tertiary referral services for complex paediatric care, including medical, surgical, trauma, and neonatal services. It admits over 10,000 children and young people to hospital each year and performs over 100,000 occasions of service through outpatient clinics [13].

## Ethical Approval

The study was approved by the Hunter New England Human Research Ethics Committee (2022/ETH00104).

## Description of the Care Practice

### Developing the Model

As part of a quality improvement initiative within HNELHD, a 0.6 FTE Project Manager and 1.0 FTE Project Officer were employed to develop and pilot the Model over a three-year period. These positions were funded via NSW state funding with the expectation that HNELHD would recruit and fund an ongoing Paediatric Care Coordinator position following a successful pilot of the Model.

Development of the Model commenced with a qualitative study in 2022 that examined the lived experience of care coordination in HNELHD from the perspectives of parents/carers of CMC and health staff working with CMC [14]. This study described the overwhelming challenges of managing complex medical needs for both staff and families, and highlighted the need for clear role delineation and communication amongst staff. This study also found that families wanted to reach a state of “equilibrium” amongst the myriad of stakeholders involved in the care of their children.

Following this study, a consultation and co-design phase was undertaken, including eight co-design workshops with 150 staff and consumer participants. The workshops took place between July and September 2022 and included representation from various roles and geographic areas. A total of 57 allied health professionals, 19 doctors, 21 managers, 38 nurses, and 15 consumers (parents, guardians, or children) attended. Fifty-five participants were from metropolitan areas and 95 were from rural and regional areas within HNELHD.

Staff were invited to participate in workshops via hospital and health district communication channels, including emails, newsletters, and printed flyers displayed in key staff areas. Participants registered their attendance through a Microsoft Teams form. As this was a quality improvement project, formal consent processes were not required.

The workshops were designed to be accessible and flexible, following a drop-in format that allowed participants to attend anytime throughout the day. Six workshops were held face-to-face at rural, regional, and metropolitan hospitals and two were conducted virtually. Upon arrival, participants checked in using a QR code and watched a brief introductory video to provide context for the project and explain the workshop structure. The workshops were organised around eight thematic stations, each featuring a scenario related to care coordination, a consumer quote, and an accompanying image.

Participants were provided with a card containing six questions to consider for each scenario:

1. What is the headline idea in five words or less?
2. What could this idea look like?
3. What are the challenges to implement this idea?
4. How might we overcome the challenges?
5. Has something similar been done elsewhere?
6. Who needs to be on board for this idea?

Participants moved freely between the stations, contributing their thoughts on sticky notes corresponding to the numbered questions. They were encouraged to build on the ideas of others or add their own suggestions, without the requirement to address every scenario or question. The workshops generated over 3,000 sticky notes, which the project team reviewed and organised using quality improvement methodology to identify actionable ideas. These ideas were assessed for feasibility and potential impact and evaluated against the strategic priorities of the district to inform the development of the Model for subsequent testing.

### Model of Care

The MoC that emerged from the workshops is comprised of two distinct phases:

1. **The Intensive Phase:** Led by a Paediatric Care Coordinator, this phase involves enrolment of eligible children into a six-to-twelve-month period of intensive care coordination support. Here, the Care Coordinator works with CMC and their families to create and initiate individualised care plans according to the specific needs of the child and family. Activities within this phase include multidisciplinary meetings, education, and coaching. Once the goals of the child and family are met, they then transition to the Maintenance Phase of the Model.
2. **The Maintenance Phase:** This phase involves reduced coordination activity with regular monitoring and review by a member of a Network of paediatric health professionals established as part of the MoC. This phase shifts from active coordination to sustained oversight and support.

**Figure 2:**
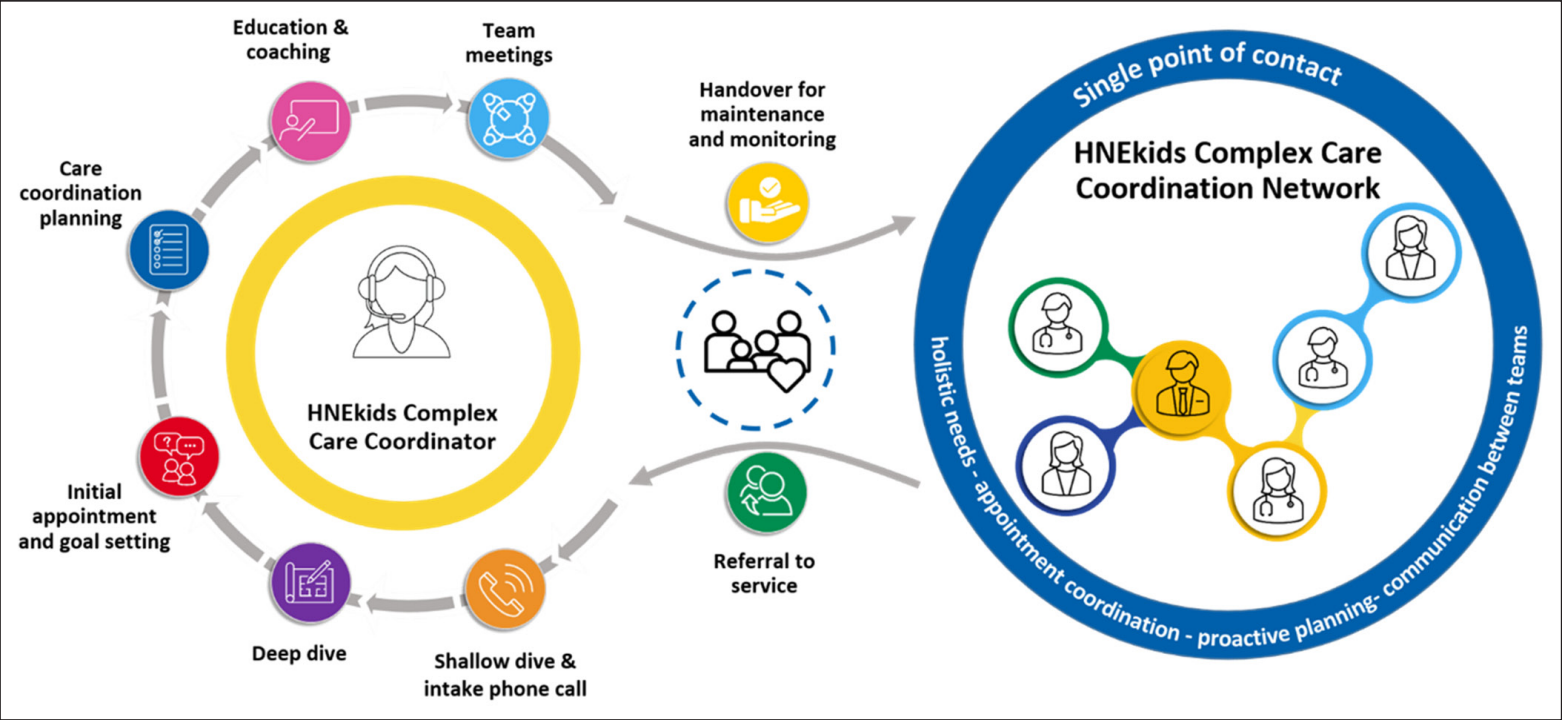
The HNEkids Complex Care Coordination Model of Care.

Figure 2 depicts the MoC and the flow of children through the Intensive Phase of care coordination and into the Maintenance Phase.

The Paediatric Care Coordinator role is a full-time position funded by the Children Young People and Families Service within HNELHD, designed to be flexible in location and open to applicants from any healthcare profession. For this implementation, the position was filled by a physiotherapist and based in Newcastle, the metropolitan centre of HNELHD.

The establishment of the Network of paediatric health professionals was led by the Project Manager and Project Officer responsible for developing the MoC. Line managers and service groups from across HNELHD were initially engaged to secure support for the Network, with discussions focused on its objectives and potential benefits to patient care. Following this engagement, 40 members were recruited to the Network, primarily comprising Clinical Nurse Consultants (CNCs) from various subspecialty teams. Additionally, the Network included an Aboriginal Health Worker, a social worker, a mental health worker, and a multicultural health worker. The primary purpose of the Network is to:

- Provide a single point of contact for families who transition from the Intensive Phase of the Model.
- Act as a Community of Practice for care coordination in HNELHD.
- Support transparency and equitable distribution of care coordination tasks across Network/team members.
- Advocate for the alignment of consistent, practical and realistic approaches to sustainable care coordination across the district.
- Exchange resources, education and evidence-based practice.

As part of Model development, a range of patient-facing guides and resources were created and made available on the Service’s website. These resources, which include information on navigating the healthcare system, understanding the care coordination process, and accessing community support services, were co-authored or reviewed by consumer representatives involved in the project. While these resources had not been formally translated into other languages at the time of publication, they were accessible on the website, making translation through tools like Google Translate readily achievable. Links to the Service page and specific sub-topics were emailed or texted to families during or shortly after their first appointment.

Establishing cross-sector connections was a key focus of the project team, with the Project Officer and Project Manager playing central roles in building these relationships. These partnerships have now been embedded within the Model, supporting their sustainability beyond the initial Implementation Phase.

To support this, a portion of the Paediatric Care Coordinator’s role is dedicated to nurturing these established relationships and fostering new ones as needed. By integrating this responsibility into the Care Coordinator’s role, the Model is designed to maintain strong cross-sector connections in the long term.

## Implementation

A 12-month pilot of the MoC commenced in April 2023. Eligible referrals (Table 1) were prioritised by the Paediatric Care Coordinator based on their vulnerability, instability, fragility, intensity, and complexity using a referral prioritisation tool (Table 2). Each criterion was weighted equally, with one point allocated to each criterion met. Children with the highest scores were ranked as needing the highest urgency of care coordination. Referrals that did not meet the eligibility criteria were not accepted into the Service. If there was any uncertainty surrounding child eligibility, the Paediatric Care Coordinator contacted the referring party directly to discuss the referral and determine eligibility.

**Table 1:** Eligibility criteria for the Model of Care.


To be eligible for the Model, children must meet all of the following criteria. Children must: Access HNEkids health services within HNELHD. Have a diagnosis expected to last greater than 12 months. Need specialty medical support under three or more specialty teams dealing with different organ systems (this can be predicted for infants). Have no existing key person already coordinating their care within a multidisciplinary team. Have the potential for a more coordinated approach in their care.


**Table 2:** Referral prioritisation tool.


PRIORITISATION CATEGORY		SCORE

**Vulnerability**	Aboriginal and/or Torres Strait Islander	

	Living in out of home care	

	Lives in a rural or regional community	

	A refugee/asylum seeker	

	Culturally and linguistically diverse background	

	Homelessness	

	Gestational age <32 weeks	

	Other	

**Instability**	Has the child had, or is expected to have, more than one emergency presentation in 12 months?	

**Fragility**	Has the child had more than 5 hospital admissions in 12 months or 30 inpatient days in 6 months? *Note: Overnight Accommodation, Day Medical and Day Surgical do not count as inpatient stays*.	

**Intensity**	Does the child have an interventional health care need and requires a technology or procedure in their home.	

**Complexity**	Has the child had greater than 20 medical appointments in 12 months? *Do not include allied health visits or any visits that could be accessed in the community but were attended at a HNELHD facility due to convenience (i.e immunisation). Appointment no can be predicated for infants*.	

	**Total score**	


The developed Model emphasised the importance of referring children who met the criteria as early as possible, ideally before they reached a point of crisis. Early referral allowed the Intensive Phase to focus on proactive care coordination, equipping families and their care teams with the tools and strategies to reduce the likelihood of future crises. However, if a family was referred during a period of crisis, the Paediatric Care Coordinator prioritised addressing their immediate needs. Once the crisis was resolved, the Paediatric Care Coordinator transitioned back to the more structured approach and cadence outlined in Figure 4 to implement long-term care coordination strategies.

**Figure 3:**
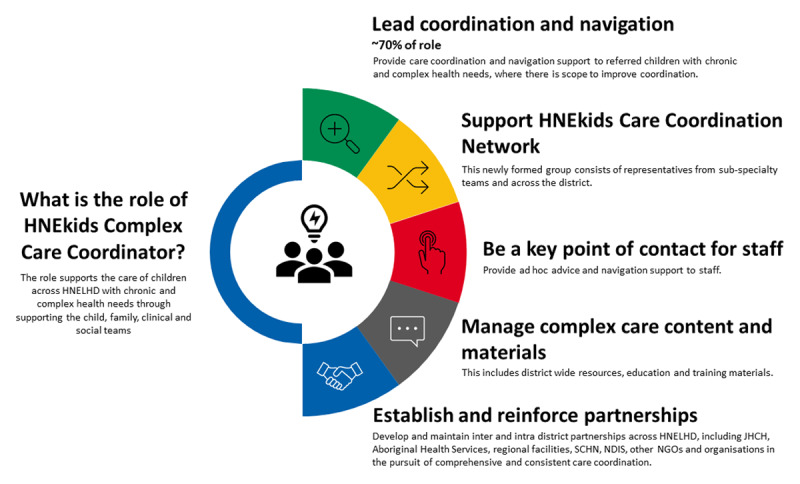
The role of the Paediatric Care Coordinator. Abbreviations used within figure: HNELHD: Hunter New England Local Health DistrictJHCH: John Hunter Children’s HospitalSCHN: Sydney Children’s Hospital NetworkNDIS: National Disability Insurance SchemeNGOs: Non-government organisations

The MoC was structured to provide comprehensive and individualised care planning for CMC. Central to this was the role of the Paediatric Care Coordinator (Figure 3), who worked in close collaboration with families to provide comprehensive care coordination and navigation support.

The Intensive Phase of the Model involved a series of virtual appointments with enrolled families to understand their situation, create an individual care coordination plan using goals of care and provide support with:

- Service coordination – facilitating communication between staff and families.
- Resource connection – linking families with relevant resources and support options.
- Documentation support – assisting with completing documents/forms associated with appointments.
- Navigation support – helping families understand and connect with services, systems and people.
- Advocacy and coaching – empowering families to coordinate aspects of their child’s care.
- Appointment coordination – assisting with streamlining appointments across multiple services.

The Paediatric Care Coordinator was provided with a laptop and phone, enabling them to work primarily through videoconferencing and phone calls to connect with families and clinical staff across the district. Although the role was designed for remote operation, being based in Newcastle allowed the Care Coordinator to meet families in person when they attended appointments at the tertiary hospital and have informal corridor conversations with clinical staff, facilitating care coordination efforts.

The Paediatric Care Coordinator primarily communicated with families through a mix of scheduled virtual appointments and phone calls. Ad hoc calls from families were generally answered throughout the day. If the Care Coordinator was unavailable, they aimed to return the call within the same day. At a minimum, a text message was sent to acknowledge the call and establish a suitable time to connect.

Family participation was a cornerstone of this Model. Families were encouraged to be actively involved in all aspects of care planning. This included attending all scheduled appointments, whether in-person or online, promptly responding to communications from the Paediatric Care Coordinator, and contributing to the planning process.

A major element of the Model was its focus on advocacy and coaching. The Paediatric Care Coordinator worked closely with the family to identify specific areas where they could benefit from coaching and support. This personalised coaching included practical guidance on how to complete transportation forms, how to request appointment changes, and how to prepare for appointments. Additionally, the Paediatric Care Coordinator helped families understand the different roles within the healthcare team.

By fostering a collaborative relationship, the Model not only supported families in managing their child’s complex care needs but also aimed to provide them with the skills and knowledge necessary to advocate for their child. This was vital for sustaining long-term engagement and equipping families to successfully coordinate care independently, even beyond the program’s direct support.

**Figure 4:**
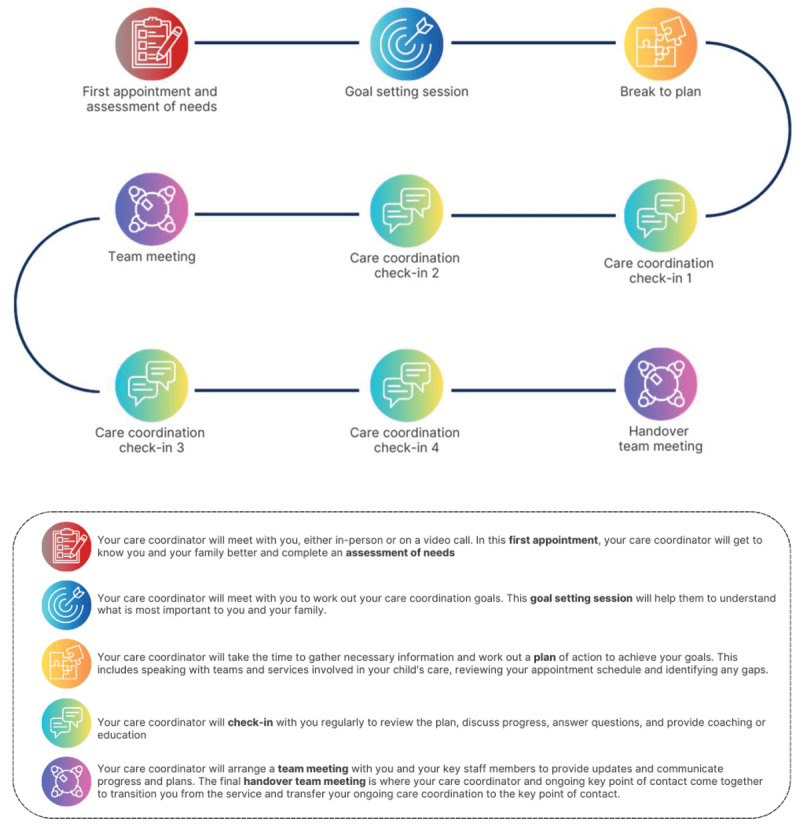
What families can expect during the Intensive Phase of the Model.

Figure 4 depicts the typical process that families experienced as they moved through the Intensive Phase of the Model.

Families enrolled in the Model remained in the Intensive Phase for six to twelve months. This included a minimum of four core check-in sessions, which provided structured opportunities to review progress, share updates, and refine goals. Between these sessions, families had ad hoc interactions with the Paediatric Care Coordinator, primarily via phone calls, to address immediate needs or concerns. As the Intensive Phase neared completion, the transition to the Maintenance Phase began. During this transition, the Paediatric Care Coordinator connected each child/family with an identified member of a Network of paediatric health professionals, who then took over as the primary contact for ongoing care coordination. This Network included 40 individual members representing key paediatric roles across subspecialty teams, facilities, and various locations within the district and adjacent districts. Network members were matched with families based on their specialty, location, and/or existing involvement with the child. In most cases, the child and family were already known to the Network member. This supported a smooth transition for the child and enabled the Network member to more efficiently and effectively incorporate care coordination tasks into their existing workload.

During the Maintenance Phase, Network members scheduled virtual or phone-based “check-in” appointments with families at intervals agreed upon by each individual Network member and family. These “check-ins” helped families remain supported post-transition and provided the opportunity to identify families who may have required re-referral to the Intensive Phase. There was no minimum timeframe for how long a child needed to be out of the Intensive Phase before being referred back in.

The Network convened virtually on a monthly basis and these meetings were chaired by the Paediatric Care Coordinator. Information sharing across the Network was supported by a dedicated Microsoft Teams channel and SharePoint site, accessible only by Network Members and the Paediatric Care Coordinator. This facilitated the secure sharing of resources and patient information across the Network and helped overcome the barrier of varying electronic medical record systems used across the district.

## Evaluation

### Methods

To evaluate the impact of the MoC, a pre- and post-implementation cohort analysis was conducted for the first 20 children who were enrolled for at least six months. Hospital utilisation data from JHCH was extracted for each child, covering the six months prior to their enrolment and the six months following their enrolment in the Model.

Collected characteristics on the children included: age, medical conditions, residential postcode, and Aboriginal or Torres Strait Islander status. Postcodes were used to classify participants as living in “urban” (i.e., major cities) or “rural” (i.e., regional or remote) areas according to the Australian Statistical Geography Standard 2021 [15]. Postcodes were also used to determine socioeconomic areas using the 2021 socioeconomic indexes for areas which were dichotomised at the NSW median into areas of ‘most disadvantage’ or ‘least disadvantage’ [16].

The outcomes examined were based on JHCH service utilisation, including the number of outpatient appointments, the number of coordinated appointments (i.e. appointments occurring on the same day), the number of inpatient stays, the cumulative length of inpatient stays (LOS), the number of did-not-attend (DNA) occurrences, and the number of emergency department (ED) presentations. Kilometres of travel were also calculated for each child as the distance between the JHCH and residential postcode. For each presentation to JHCH, this distance was multiplied by two to represent travel to and from the facility.

Additionally, ED presentation data for each child was obtained from JHCH and four major regional hospitals within the HNELHD.

Due to the small sample size (n = 20) and non-normal distribution of the data, non-parametric testing was used to determine the statistical significance of the differences between each pre and post implementation outcome [17]. The Sign test was used to determine if pre-post differences for the entire cohort were significantly different from zero. Wilcoxon rank-sum tests were used to examine the association between pre-post differences and residential rurality.

To avoid data skew, neonatal intensive care unit (NICU) inpatient data was excluded from analysis, as NICU events are typically longer in duration and early in occurrence.

The Normalization Measure Development (NoMAD) survey was used to assess how well the new Model had been integrated into routine workflows. Based on Normalisation Process Theory (NPT), the survey evaluates the mechanisms by which new interventions become embedded and sustained in everyday practice [18]. In addition to the NoMAD questions, the anonymised survey included a free-text space for respondents to provide additional comments regarding the new Model. The survey was conducted between March and May 2024, approximately 12 months after the Model’s introduction. It was distributed through a Microsoft Teams Form as part of normal quality improvement project process, and no formal consent was required. The survey was sent to staff within the Network and those who had referred into the service.

A total of 47 staff responded to the survey, including 22 nurses (47%), 18 allied health professionals (38%), 5 doctors (11%), and 2 managers (4%). Of these respondents, 27 (57%) worked at JHCH, while the remaining 20 (43%) were from regional hospitals within the district.

Informal feedback was gathered from families during routine service engagement by the Project Officer, as part of the quality improvement process. Of the 20 families who had been enrolled in the Model for at least six months, all were contacted by phone and asked to provide feedback on their experience with the service. Five families provided verbal consent for their anonymised feedback to be included in written reports and presentations. These brief, free-form conversations focused on how families felt about the Model and were conducted as part of standard consumer input for routine quality improvement purposes.

### Findings

#### Population Study Characteristics

Between April 2023 and March 2024, a total of 44 children were referred into the Model. Of these, 39 were accepted. The reasons for not accepting the remaining five referrals included the presence of an existing role already coordinating care for the child in four cases and a parent declining enrolment in one case.

The average age of children at the time of referral was 3 years, with ages ranging from 2 months to 15 years. These children had a broad range of conditions, including rare genetic, hormonal, and metabolic syndromes, reflecting the heterogeneity of the referred population.

Children enrolled in the Model typically saw an average of 31 individual clinicians under eight separate specialties at the tertiary paediatric hospital. These figures represent just a fraction of their care landscape, as many also required support from a myriad of healthcare, disability, and social support professionals outside the tertiary setting.

Of the 39 children enrolled in the Model, 20 were enrolled early enough to be included in the pre-post analysis, as they had participated in the Model for at least six months.

The 20 enrolled children exhibited additional sociodemographic vulnerabilities, further complicating their care needs. Forty percent (n = 8) of children identified as Aboriginal or Torres Strait Islander. Five percent (n = 1) were from culturally and linguistically diverse (CALD) families requiring interpreter services. Seventy percent (n = 14) resided in rural or remote areas of HNELHD, while 30% (n = 6) lived in metropolitan areas. Ninety percent (n = 18) resided in socioeconomically disadvantaged areas (IRSAD decile ≤5), and 70% (n = 14) required social worker intervention. Of the 20 children, only two had transitioned to the Maintenance Phase at the time of analysis. Neither child required re-entry into the Intensive Phase, although ongoing monitoring will determine whether this becomes a factor over time. The remaining children were still within the Intensive Phase but were on track to transition to the Maintenance Phase within 12 months of their enrolment.

#### Impact on hospital utilisation and appointment coordination

A total of 153 outpatient appointments were attended by the cohort (n = 20) in the six months prior to enrolment, and a total of 139 were attended in the six months post enrolment.

In the six months prior to enrolment into the Model, 10% (n = 2) of children experienced coordinated outpatient appointments on the same day at JHCH. Six months post enrolment, this figure increased to 65% (n = 13). Children saw a median increase of 2 coordinated appointments over time (p = 0.002). Across the entire sample of 20 children, this corresponded to an increase of 4 (pre) to 49 (post) total appointments held on the same day. While initially these coordinated appointments constituted only 2.61% of the total appointments attended by the cohort, they now account for 35.25% of all appointments.

A total of 37 ED presentations were recorded for the cohort in the six-months before enrolment, with 55% (n = 11) of the cohort presenting at least once. In the six-months post-enrolment, the total number of ED presentations dropped to 14, with 40% (n = 8) of the cohort presenting at least once. However, the pre-post difference in ED presentations per child was not found to be statistically significant (p = 0.27). The location of ED presentations remained consistent pre- and post-enrolment, indicating no change in where children sought emergency care.

In the six months prior to enrolment, 60% (n = 12) of the cohort was admitted as an inpatient for at least one day, with a range of 1 to 5 admissions per child. The LOS per admission ranged from 1 to 136 days, totalling 276 days. In the six months following enrolment, 30% (n = 6) of the cohort was admitted as an inpatient for at least one day, with a range of 1 to 9 admissions per child. The LOS per admission ranged from 1 to 27 days, totalling 110 days. When comparing pre and post enrolment data, a non-significant median difference in LOS of –0.5 days was observed (p = 0.09).

The overall difference in the number of inpatient stays across the cohort was not found to be statistically significant (p = 0.11), with a mean of 1.15 admissions per child pre-enrolment and 1.2 per child post-enrolment. No significant difference was found either between pre-post DNA occurrences in the Model.

No significant differences in any measure of pre-post hospital utilisation were identified between children residing in urban or rural areas.

#### Impact for families of CMC

Over 60% of children enrolled in the Model travel away from their hometown for outpatient appointments at JHCH. Of these, 50% travel more than 200 kilometres to reach JHCH. Based on the observed increase in coordinated appointments and reduction in ED presentations for the cohort, an average of 303 km and median of 88 km of travel was saved per family in the six months following enrolment (p < 0.001). This can be translated to an anticipated average saving of 606 kms per family, each year. Where children reside rurally, this saving increases to 1000 kms on average per family, each year.

As part of routine quality improvement activities, families were asked to provide feedback on their experience with the Model. Families described the significant mental load they faced before enrolment, including juggling their child’s care alongside other responsibilities such as work and family life. Many shared that the support of the Paediatric Care Coordinator gave them the space to pause, regroup, and better organise their child’s care. Families felt more equipped to face challenges beyond the Intensive Phase, though this was not formally evaluated. This sense of relief and support was described as invaluable and has been referred to as “coordination respite,” reflecting the unique relief families experienced during their time in the Model.

#### Acceptability of the Model from healthcare providers

The NoMAD Survey was completed by 47 health professionals working with CMC in HNELHD (78% response rate). 83% of respondents acknowledged a shared understanding of the purpose of the MoC, and 87% could clearly distinguish the Model from usual practices, underlining its innovative nature.

**Figure 5:**
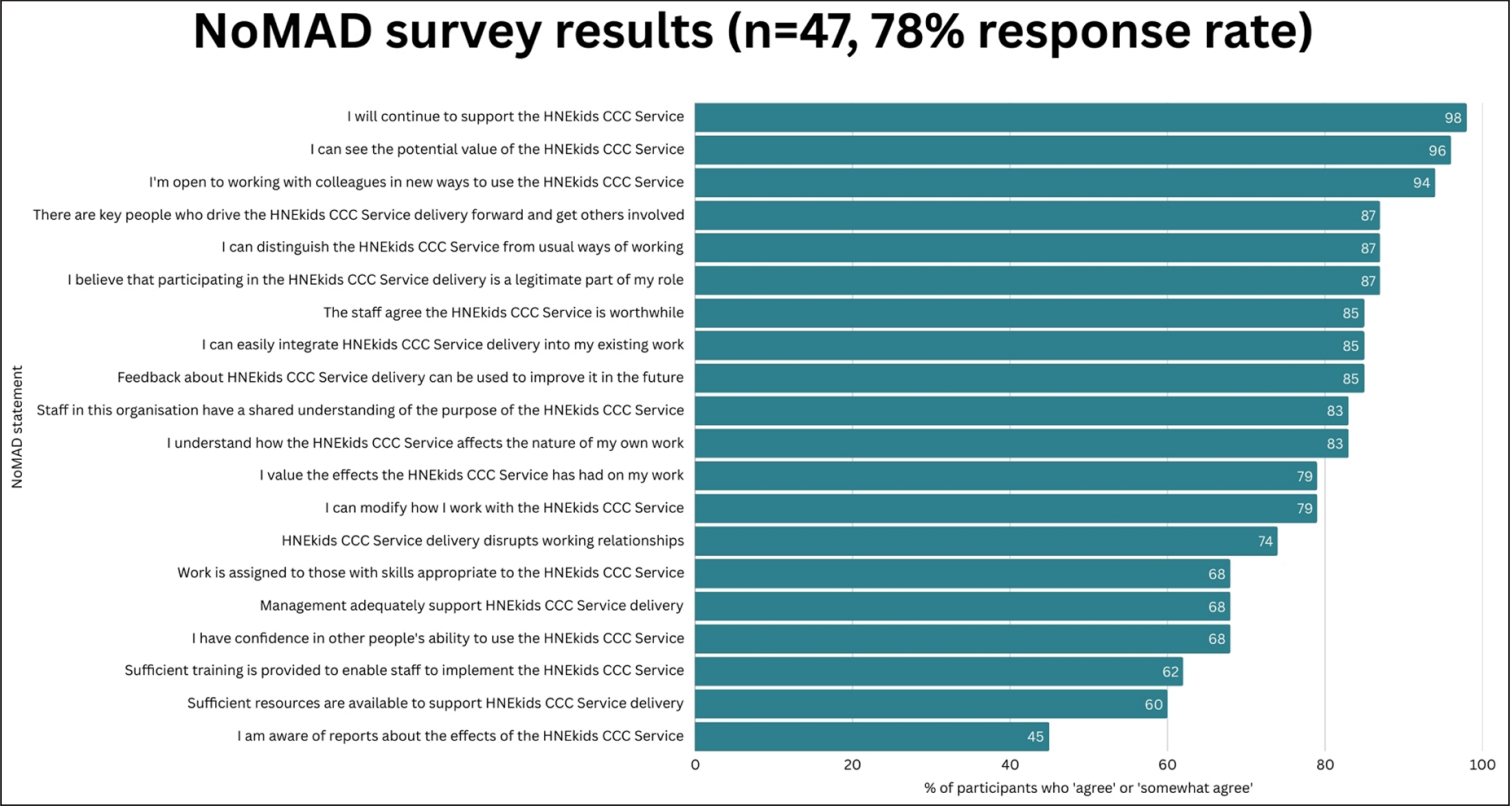
Results of Normalization Measure Development (NoMAD) Survey. Abbreviations used in figure: HNEkids CCC Service: HNEkids Complex Care Coordination Service

Further, 96% of participants recognised the value of the Model, and 87% affirmed that engaging in the delivery of the Model aligns with their professional roles, indicating strong internal support for the initiative’s objectives. The ability to integrate the Model into existing workflows was highlighted by many respondents (85%). See Figure 5 for full survey results.

Examples of additional written feedback provided by health staff are included in Table 3.

**Table 3:** Written feedback from health staff.


*“This is a worthwhile service for our kids and families.”* *“This service has been invaluable for the care of my complex patients who otherwise get lost amongst the many services they access within healthcare.”* *“The Network has been a great source of new info for me. It has made coordinating care easier for the kids I look after – even if they haven’t gone through the actual intensive care coordination with the Care Coordinator.”* *“I can already see how this service is helping to make clear who is doing what. It’s accountability, plus support. And I can see it is sustainable. It’s great to be part of the solution!”*


## Discussion

This study presents the early implementation results of a short-term Care Coordination Model for CMC. The significance of this study lies in its description of a Model that incorporates an intensive “dose” of care coordination, while simultaneously applying a Network approach to leverage the expertise and structure of the existing system and sub-specialty workforce. The early results, though limited in scale and focusing predominantly on the Intensive Phase of the Model, indicate a more efficient use of hospital resources, benefits to the enrolled families, and acceptability by staff.

The co-design process was central to the development of the Model. The project embraced co-design principles [19,20,21,22] by collaborating with families, healthcare professionals, and community representatives. Consumers were involved from the outset of the project, co-designing evaluation measures, communications, recruitment, and ethics protocols. This inclusive approach meant that the Model was practical and aligned with the needs of both families and healthcare providers.

The two-tiered approach to care coordination in our Model begins with an Intensive Phase where families receive focused support, goal setting, and comprehensive care coordination to address their immediate needs. Following this, the Maintenance Phase involves a representative from a newly established Care Coordination Network providing structured ongoing monitoring and support to families. This Network approach unites existing roles into a Community of Practice, reducing duplication of efforts and offering a single point of contact for families beyond the Intensive Phase. By clarifying responsibilities and assigning children to specific individuals within the care team, this approach is designed to minimise missed or duplicated work. Established in response to staff feedback about the lack of clarity in care coordination roles and responsibilities, this Network is set to provide continuity and sustained support for children transitioning from the Intensive Phase, reducing the risk of children slipping through the cracks. To our knowledge, this specific two-tiered approach is not discussed in existing research, but it is supported by evidence highlighting the importance of building workforce capacity for complex care coordination and the positive impact of Communities of Practice [23,24].

Investigating the optimal dose of care coordination for CMC is important for creating value-based care models. Previous studies have indicated that continuous care coordination, while beneficial, can be challenging to maintain due to its cost-intensive nature [25]. By determining the optimal dosage of care coordination, healthcare providers can support CMC to receive the appropriate level of care to meet their unique needs. This approach aligns with value-based care principles, which aim to deliver the best outcomes at the lowest cost [26]. Implementing a time-limited, Intensive Phase of care coordination allows for focused intervention when families need it most, followed by a Maintenance Phase of sustained support without overwhelming resources. This Model not only addresses immediate healthcare needs but also aims to empower families to manage care independently in the long term, potentially reducing overall healthcare utilisation and costs. Additionally, the concept of “coordination respite,” a term developed during our project, highlights the significant relief families experience when provided with structured, time-limited support. This respite not only alleviates the immediate pressures faced by families but also restores their capacity to effectively engage in their child’s care, further emphasising the value of a well-calibrated care coordination model.

The Intensive Phase of the Model significantly increased the coordination of same day appointments. Coordinated appointments reduce travel, associated out-of-pocket expenses, and school and work absenteeism [27]. For families residing in rural and remote areas or those facing socioeconomic challenges, these savings are especially significant. These findings are supported by other research, which highlights the benefits of coordinated care in reducing financial, logistical and other strains on families [28,29,30,31].

In addition to enhancing the healthcare experience for children and their families, appointment coordination may enable more efficient and effective use of hospital resources [32]. Health staff involved in this study reported that same-day appointments reduced the need for repeat visits, procedures, and tests [33]. This approach also facilitated opportunistic immunisations and allowed multiple procedures to be performed under a single anaesthetic [34]. Consequently, hospital expenditure on administrative processing and the provision of hospital-based patient/family subsidies and services can be reduced.

Family empowerment is a core focus of the Model. Families were given the tools and support to navigate the healthcare system more effectively, which included goal setting, team communication, appointment and transport bookings, paperwork assistance, and understanding the health system. The resources provided to families were co-designed and co-authored with consumers. This approach aligns with previous research about building the capacity of families to navigate complex healthcare systems and to enable self-management where possible to assist in managing service demand [35].

The health of CMC is closely linked to the well-being of their families [4]. Adverse social determinants of health (SDHs) and adverse childhood experiences are associated with poorer health outcomes for children [36,37]. The impact on caregivers is substantial, including chronic sleep issues, reduced time with partners and siblings, limited personal care, deteriorating mental health, and lower overall quality of life [29,38,39,40,41]. Caregiver stress may hinder the execution of necessary medical care for CMC, underscoring the critical nature of this issue [42]. Without targeted social work support, barriers such as socioeconomic status and language differences may deepen existing disparities, compromising care quality and safety, and increasing family and provider strain [19,43,44,45,46]. Integrating psychosocial elements and social work within future Care Coordination Models is therefore recommended as essential for addressing these risks and improving health outcomes for CMC.

### Limitations of the evaluation

Within the evaluation timeframe, only two children had transitioned into the Maintenance Phase, limiting the evaluation to the short-term impacts of the Intensive Phase of the Model. Longitudinal data is needed to assess the effectiveness of the Maintenance Phase and the overall impact of the Model over time.

While hospital utilisation data showed a decrease in service use among enrolled families, the absence of a control group in the evaluation limits the ability to determine whether the observed changes were influenced by other factors.

The small sample size of 20 children further limits the generalisability of the findings to the broader population of CMC. Larger cohorts in future evaluations will be necessary to strengthen the reliability of these results.

Additionally, the reasons why some families had not transitioned to the Maintenance Phase after six months of enrolment were not examined in this study. Future evaluations should explore whether psychosocial factors, potentially addressable through the support of a dedicated social worker, may contribute to prolonged reliance on the Intensive Phase.

## Lessons learned

- **Optimal dose of care coordination:** Determining the optimal duration and intensity of care coordination is an ongoing process. Early findings suggest that families can transition from an Intensive Phase to a Maintenance Phase within 6–12 months, providing a foundation for further evaluation of this approach to support sustainability and scalability.
- **Family empowerment:** Building the capacity of families to navigate complex healthcare systems is vital. Providing tools, resources, and support enables families to manage their child’s care more effectively, reducing their reliance on healthcare providers and enhancing their ability to advocate for their child’s needs.
- **Network approach:** Establishing a Network of paediatric professionals across various specialties and geographic locations promotes cohesive care strategies and robust connections among care providers.
- **Information sharing:** Variations in clinical information systems across HNELHD created challenges for communication. Microsoft Teams and SharePoint were key tools that supported effective collaboration and information sharing among the Care Coordination Network and the Paediatric Care Coordinator.
- **Psychosocial complexity:** Piloting the Model highlighted the significant psychosocial needs of enrolled families, many of whom faced challenges such as financial hardship, social isolation, and difficulty accessing community resources. While the Paediatric Care Coordinator was able to assist with some of these issues, the demand and complexity of many situations exceeded the scope of their role. Addressing these needs often required referrals to general social work departments, which were frequently delayed due to waitlists. Future iterations of the Model should consider integrating a dedicated social worker to manage these needs more effectively and in a timely manner.
- **Importance of defining role scope:** The development of the Model highlighted variability in how the term “care coordination” is understood by different stakeholders. Clearly defining the scope of the Paediatric Care Coordinator’s role and the responsibilities of the Care Coordination Network was crucial for fostering a shared understanding of the Model and aligning expectations among staff, referrers, and enrolled families.

## Conclusion

By incorporating an intensive, time-limited phase of care coordination followed by a maintenance phase supported by a Network of paediatric professionals, this Model addresses the unique needs of CMC and their families. Early results indicate improvements in operational efficiency, reductions in hospital resource utilisation, and enhanced family satisfaction. Moreover, the Model has shown high acceptability among healthcare staff, demonstrating strong internal support and integration into existing workflows. As the Model transitions into standard practice, ongoing evaluation of the Model as a whole will be crucial to refine and sustain its benefits, ensuring that it continues to provide value-based, family-centred care in the long-term.
